# A Survey Into Child and Adolescent Mental Health (CAMH) Staff Wellbeing Within a Trust in England

**DOI:** 10.1192/bjo.2024.514

**Published:** 2024-08-01

**Authors:** Iqra Shamim, Reka Ajay Sundhar, Kiran Panesar

**Affiliations:** Black Country Health Care Trust, Birmingham, United Kingdom

## Abstract

**Aims:**

Sickness absences within the NHS have been on the rise with 27 million days across 2022, 22% of which were due to mental health and wellbeing related issues. The 2022–23 report on progress in improving NHS mental health services found that increased workload and staff shortages within the mental health sector was leading to concerns of staff ‘burnout’ and higher rate of staff turnover. There is an indication that CAMHs staff are at higher risk of poor wellbeing despite limited research in the UK. The audit aimed to gain insight into the wellbeing of staff working in CAMHs within the trust according to NICE Mental Wellbeing at Work (NG212) and highlight areas of concern and propose recommendations to improve staff wellbeing.

**Methods:**

Anonymous self-report questionnaire, based on a validated tool recommended in the NICE NG212 guidance, was disseminated across four boroughs within the trust to measure wellbeing in the workplace. The survey had 19 questions, 17 requiring scores on Likert scales and 2 descriptive questions, aimed to gain information on the five drivers of wellbeing in the workplace (health, relationships, environment, security, and purpose).

**Results:**

A total of 123 responses were analysed. Across all four boroughs, physical health scored worse than mental health. 44.7% of respondents felt they had inadequate facilities in the workplace to support their health and wellbeing. Further, 32.5% of responses disagreed/strongly disagreed that change was managed well in their organisation compared with 25% who agree/strongly agree. Key words such as staff-appreciation and team-building days recurred in qualitative responses across the trust. There were further site-specific differences. Research shows that provision of workplace exercises can be safely used for the promotion of employees' physical and mental health. Cost-effective strategies such as staff-appreciation and gratitude can improve job satisfaction thereby promoting staff retention. To consider elements of face-to-face contact with colleagues could also have a positive impact on psychological wellbeing due to more productive meetings; increased networking; increase of engagement and job satisfaction.

**Conclusion:**

The CAMHS staff wellbeing survey provided useful insight into staff perception of their wellbeing at work. Repeating the survey, after implementing recommendations, would help identify important determinants. Despite the survey being limited to the specific trust, further research into CAMHs staff wellbeing across the UK could help facilitate improvements and help with staff retention.

